# Cross Jurisdictional Boundaries to Build a Health Coalition: A Kentucky Case Study

**DOI:** 10.3389/fpubh.2018.00189

**Published:** 2018-07-10

**Authors:** Angela L. Carman, Margaret L. McGladrey

**Affiliations:** ^1^College of Public Health, University of Kentucky, Lexington, KY, United States; ^2^Tisch College of Civic Life and Department of Sociology, Tufts University, Medford, MA, United States

**Keywords:** cross jurisdictional, public health, community, assessment, improvement

## Abstract

Cross-jurisdictional sharing is accomplished through collaboration across jurisdictional boundaries to deliver essential public health services and solve problems that cannot be easily addressed by single organizations or jurisdictions. Partners across 10 counties and three public health jurisdictions of the Barren River Area Development District (BRADD) convened as Barren River Initiative to Get Healthy Together (BRIGHT), a community health improvement coalition. Focus groups and interviews with BRIGHT members indicate that the use of effective strategies to focus collaborative health improvement efforts fosters a cohesive coalition even when the group is populated by individuals from across public health jurisdictional boundaries. Focusing strategies identified included: the importance of organizing workgroups so members can draw upon expertise, adoption of a community engagement model for health assessment and improvement; and use of a facilitator, who offers guidance and administrative support to groups and focuses members on accomplishing goals.

## Introduction

America's Health Rankings provides an overall ranking for each state based on a number of health factors, such as behaviors, clinical care, and environment, and health outcomes, such as cancer and cardiovascular deaths. In the 2017, America's health ranking listed Kentucky 42nd among the 50 states (1). This ranking is but one of many sources indicating the urgent and immense need for health improvement in the state (2, 3).

A potential catalyst for large-scale health improvements in Kentucky may be its local public health departments (LHD), as Kentucky ranks among the top states with LHDs accredited by the Public Health Accreditation Board (PHAB) (4). These accredited health departments and those pursuing national, voluntary public health accreditation follow a system of standards and measures are created in domains that mirror the 10 essential public health services (EPHS), a framework developed by the Core Public Health Functions Steering Committee (5, 6) Specifically, EPHS #4 directs LHDs to “mobilize community partnerships and action to identify and solve health problems;” (5) therefore, LHDs often serve as conveners of community partners, such as hospitals, schools, non-profit organizations, businesses, faith-based organizations, and others whose objectives include health improvement (7).

## Description of the case

**Figure 1:**
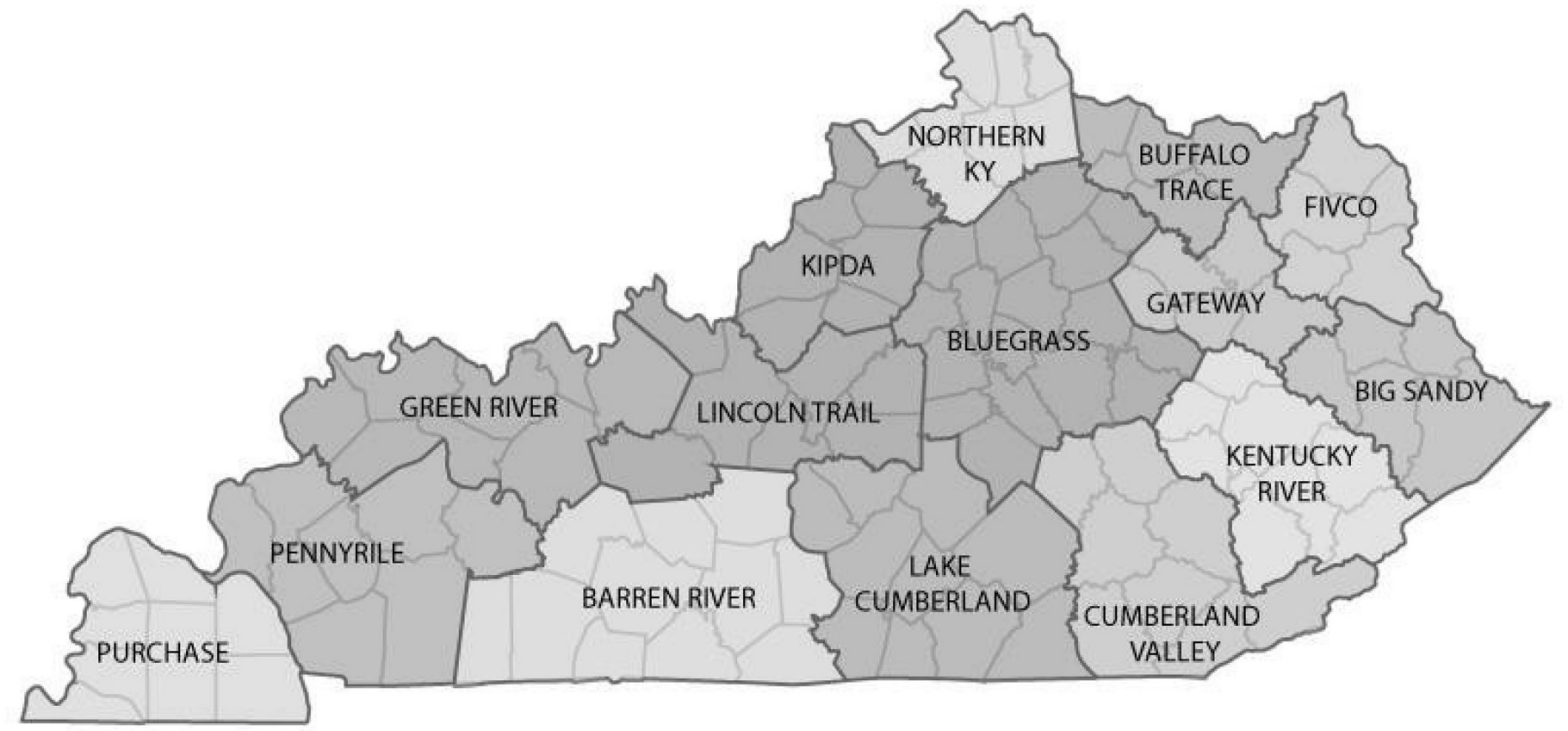
Ten counties of the Barren River Area Development District. (https://www.kaedonline.org/resources/kentucky-area-development-districts/).

In Kentucky, the local governmental public health system includes county-level health departments in all 120 counties. These county-level health departments are configured into 61 jurisdictions; 14 are multi-county districts, and 47 are single-county jurisdictions. The Barren River Area Development District (BRADD; see Figure 1) is located in southern Kentucky and includes 10 counties and three public health jurisdictions. The Barren River District Health Department jurisdiction comprises eight county-level health departments, all of which are managed from a central district office with one district director, a district board of health, and 8 local boards of health, one for each county (8). The remaining two 66 counties of the BRADD are single-county public health jurisdictions, each with their own public health director and local board of health (9, 10). These three public health jurisdictions engage with a wide variety of community partners: 10 county-level school systems, eight hospitals, businesses, non-profit organizations, county and city governments, and many others (11). In addition, the BRADD counties vary in population size, educational levels, median household income, and access to primary care providers (see Table 1).

**Table 1:** Comparison of Barren River Area Development District Counties (BRADD).

**Counties of BRADD**	Population^*^	Bachelor's Degrees or Higher (% of people 25 years+)^*^	Adult Prevalence of Smoking (% Age- adjusted)^**^	**Lung, trachea & bronchus cancer incidence (rate per 100,000, age-adjusted)^∧^**	**Teen-Birth Rate (ages 15-19; rate per 100,000)^∧∧^**	Median Household Income ^***^	**Primary Care Providers (per 100,000)^∧∧∧^**	**Public Health Jurisdiction Type**
Allen	20,640	13.6%	31.9%	63.6	51.8	$42,287	9.8	Single- County
Barren	43,148	15.6%	29.0%	106.0	51	$40,017	106.9	District
Butler	12,875	8.7%	17.0%	80.3	46.3	$39,545	7.8	District
Edmonson	12,161	13.3%	30.4%	105.5	31.5	$38,673	8.3	District
Hart	18,597	10.3%	29.4%	117	54.9	$34,764	43.1	District
Metcalf	9,990	11.6%	36.7%	104.2	73.3	$32.654	10.0	District
Logan	26,867	13.2%	30.9%	109.8	48.1	$43,795	40	District
Monroe	10,667	14.1%	25.4%	106.8	51.1	$31,603	56.2	Single- County
Simpson	17,826	15.1%	33.7%	122.7	51.5	$45,269	39.3	District
Warren	120,460	28.1%	26.2%	93.8	22.7	$48,925	82.8	District

* *US Census 2011-2015*.

** *Behavioral Risk Factor Surveillance System (2006-2012)*.

∧ *SEER 2008-2012*.

∧∧ *National Vital Statistics System 2011-2013*.

*** *Small Area Income & Poverty Estimates*.

∧∧∧ *Area Health Resource File*.

## Background and rationale

Accomplishing goals like community health improvement that involve convening community organizations across jurisdictions can be complex. Organizations may serve similar populations within a Kentucky county but have very different goals and funding sources (12). In addition, LHDs seeking to mobilize their community partners around health improvement initiatives have limited resources with which to accomplish their increasing responsibilities (13–15). These internal LHD resource constraints are compounded by the challenge of organizing health improvement efforts with community organizations distributed across geographically isolated rural areas.

Cross-jurisdictional sharing is “the deliberate exercise of public authority to enable collaboration across jurisdictional boundaries to deliver essential public health services and solve problems that cannot be easily solved by single organizations or jurisdictions”(16). Researchers have studied the extent of cross-jurisdictional sharing among LHDs; (17–20) however, despite the need for efficient use of LHD resources (13–15), little is known about cross-jurisdictional sharing in community health improvement efforts.

This study is novel in that it leverages the local expertise of BRADD community partners in the Barren River Initiative to Get Healthy Together (BRIGHT) whose resource constraints and geographic isolation make coalition work complex to identify how and why rural partners across the 10 counties and three local public health jurisdictions convened as a health improvement coalition. It also explores how community partners sustained their efforts by assessing health needs and developing and implementing cross-jurisdictional community health initiatives to address those needs.

## Methods

Identification of characteristics and activities of the BRIGHT coalition that supported cross-jurisdictional community health improvement was completed in two phases: a focus group with the BRIGHT coalition and semi-structured interviews with key informants who were identified by their peers as representing the coalition's four workgroups (Healthcare, Schools, Worksites, and Community). The study was approved by the University of Kentucky's Institutional Review Board.

### Focus group

Researchers attended a meeting of the BRIGHT coalition to assess the group's membership (*n* = 80). The first element of the assessment divided the membership into the coalition's four pre-established workgroups. The coalition developed these workgroups from the areas of expertise represented in the larger group that the coalition found to be relevant to its efforts: healthcare, worksites, schools, and other community resources. Each group discussed the question “Why did you come together with the BRIGHT coalition?” Researchers captured a total of 101 responses.

The second element of the assessment divided coalition members into dyads to discuss the question “What kept you coming back to the BRIGHT meetings?” Each dyad participated in a nominal group technique through which 93 responses were captured and grouped by theme.

### Interviews

Researchers asked coalition members to nominate individuals from each workgroup (Healthcare, Worksites, Schools, and Community) to participate in semi-structured interviews to elaborate focus group findings. A purposive sampling strategy resulted in interviews conducted with three members of the Healthcare workgroup, one Worksite workgroup member, one Schools workgroup member, and two Community workgroup members, for a total of seven interviews. Interview questions elicited detailed descriptions of why the interviewee became involved with BRIGHT, how BRIGHT operates as a health improvement coalition, and why the key informant continues to be involved in the group. Interview responses were recorded, transcribed, and thematically analyzed.

### Analysis

The focus group responses to each open-ended question were analyzed for recurring themes. Interview transcripts were analyzed using constant-comparative techniques (21) to trace their continuity with focus group themes explaining initial involvement in BRIGHT and to identify those elements of BRIGHT's operation that were seen as valuable in achieving the purpose of the group. In addition, the analysis assessed how interview and focus group data converged regarding the question of why group participation continued.

## Results and discussion

Coalition members identified five overarching reasons for joining BRIGHT during the focus group session. The ability to collaborate and network with the stakeholders represented in the coalition was the most frequently stated reason (*n* = 31), followed by a desire to improve health (*n* = 24) and to communicate, educate, or promote health- and wellness-related information (*n* = 15). A number of participants indicated that the mission of the BRIGHT coalition had a direct connection to their individual or organizational job responsibilities (*n* = 14). The remaining reasons included a desire to assess community health-related needs and resources (*n* = 8) and an individual invitation or pre-existing positive relationship with the individual seen as the convener of the coalition (*n* = 8).

We identified three primary reasons why members kept returning to the BRIGHT meetings. Consistent with their reason for joining the coalition, the opportunity to collaborate, and network with stakeholders was the most frequently stated reason for continued participation (*n* = 36). A close second was a sense of accomplishment, progress, and ability to “get the job done” (*n* = 32). One respondent stated that BRIGHT allowed the group to “accomplish things we couldn't have done on our own.” Respondents also indicated that BRIGHT meetings were organized, had excellent leadership, and held members accountable for tasks (*n* = 18).

Interview participants revealed greater detail about how and why the BRIGHT coalition works. Participants shared sentiments such as, “We are a coalition, we are working across our silos but in our comfort zones at the same time.” This participant elaborated that the coalition as a whole broke down silos by bringing together partners from different disciplines, while the workgroups allowed members from the same types of organizations (e.g., businesses, schools) to collaborate. Another participant said, “The group represents an informed, key advocacy group in their respective circle of influence for 160 improved health status in their community.” For this informant, coalition workgroups provided opportunities for members of circles of influence to convene and coordinate. The common theme among interview participants was that the workgroups organized and focused these circles of influence. One participant said, “I think the groups give focus. The work is so big. Without the groups, there would be so many directions to go.”

When probed to explain the purpose of the BRIGHT group, interview participants converged on a common theme of improving health by creating the best quality of life as well as by promoting healthy lifestyles and improved health status. Reasons given for their individual commitment to BRIGHT's purpose mirrored those of the larger coalition, including aligned job responsibilities, collaboration, and relationships with the convener. Participants also were asked to discuss the challenges of involvement in the coalition. Examples were shared of the time-consuming nature of coalition work and the need to coordinate participation with their daily personal and professional responsibilities.

Interview participants were asked about operational characteristics of BRIGHT, such as its use of a specific community health assessment and improvement model, Mobilizing Action through Planning and Partnership (MAPP) (22), to guide its work. Interview participants described the model as a step-by-step process, roadmap, framework, and evidence-based format. One participant said the model provided a “clear visual for people to understand where you're starting and what the ultimate goal is.”

Participants described working with the convening organization, Barren River District Health Department (BRDHD). The role of BRDHD in the BRIGHT coalition was defined as that of a facilitator: bringing people together, organizing and distributing information, hosting meetings, and completing other administrative tasks. BRIGHT members described BRDHD as the ideal convener; respondents said, “They created a safe space for us to be creative and try to find solutions; very open to ideas. They push us, which is good. It keeps us on task” and “We couldn't have done it without them; absolutely key.”

## Lessons learned and recommendations

In the case of the 10 counties of the BRADD, a wide variety of partners must dedicate their efforts to identify and sustain health improvement initiatives. The experiences of BRIGHT members demonstrate that employing appropriate strategies to focus collaborative health improvement efforts can create a cohesive coalition even when the group comprises individuals from across public health jurisdictional boundaries. Focusing strategies included:

### Drawing upon expertise

Key lessons included the importance of convening stakeholders who not only represent organizations that can impact health but also are open to sharing information and learning together. Organizing the large cross-jurisdictional group into smaller workgroups that draw upon the respective areas of member expertise creates a productive venue for focused discussion. The structure of these workgroups (Healthcare, Worksites, Schools and Community) also might provide a model for future projects to implement targeted health improvement interventions. For example, the larger coalition might determine that obesity is an issue of great importance across jurisdictional boundaries, and the School workgroup could identify an evidence-based practice to prevent obesity in school-age children and disseminate that practice to each school across all counties and city schools involved in the coalition.

### Use a model to provide direction and focus

The participants in BRIGHT employed systems to provide focused direction to health improvement activities. BRIGHT's adoption of MAPP as an evidence-based community engagement model for health assessment and improvement gave the coalition a direction, a series of steps, and the support of stakeholder groups to guide their work. The coalition's collective efforts to plan their health improvement activities using a well-established model produced a shared vision and mission orienting the coalition's work that transcended jurisdictional boundaries.

### Facilitate collaborative movement toward accomplishment

Leaders from BRDHD were described as organized, excellent communicators, champions for health improvement, open to new ideas, and good listeners who are able to keep the group on task. These are the core elements of the facilitator role, which offers guidance and administrative support to groups while focusing them on accomplishing goals. The leadership successes of the BRIGHT coalition demonstrate that a strong facilitator function is essential to community health improvement coalitions that involve cross-jurisdictional sharing.

## Limitations

One limitation of the analysis was the lack of focus group attendees or semi-structured interviews from those who no longer participate in BRIGHT. Information from these individuals and organizations could have provided a different perspective on the evolution of BRIGHT and identified ways to improve retention. The findings of this case study may not be generalizable to other cross-jurisdictional efforts or similar community coalition settings. In addition, this study focuses on the process of coalition development and sustainability and does not assess the impact of the coalition on health outcomes.

## Conclusion

Despite the complexity of convening stakeholders across 10 counties and three public health jurisdictions, the lessons learned from the community partners contributing to this case study are ironically not cross-jurisdictionally focused. The practice of subdividing the larger coalition into workgroups that draw upon members' areas of expertise and circles of influence, the value of a focusing model, and the importance of a facilitator to provide organization and administrative support are lessons applicable to many multi-stakeholder efforts. These lessons also add value to cross-jurisdictional groups, specifically by emphasizing the “how” of convening and sustaining members who, due to geographic or jurisdictional barriers, may have little knowledge of each other as individuals, yet deeply understand each other's circles of influence (Healthcare, Worksites, Schools, and Community) and the potential for impact that working together makes possible.

## Author contributions

AC and MM made substantial contributions to the conception and design of the work, acquisition and analysis of data, drafting, and revising the manuscript and giving final approval of the manuscript.

### Conflict of interest statement

The authors declare that the research was conducted in the absence of any commercial or financial relationships that could be construed as a potential conflict of interest.
